# Plot-level rapid screening for photosynthetic parameters using proximal hyperspectral imaging

**DOI:** 10.1093/jxb/eraa068

**Published:** 2020-02-24

**Authors:** Katherine Meacham-Hensold, Peng Fu, Jin Wu, Shawn Serbin, Christopher M Montes, Elizabeth Ainsworth, Kaiyu Guan, Evan Dracup, Taylor Pederson, Steven Driever, Carl Bernacchi

**Affiliations:** 1 Department of Plant Biology, University of Illinois at Urbana-Champaign, Champaign, IL, USA; 2 Carl R. Woese Institute for Genomic Biology, University of Illinois at Urbana-Champaign, Champaign, IL, USA; 3 Environmental & Climate Science Department, Brookhaven National Laboratory, Upton, New York, USA; 4 School of Biological Sciences, University of Hong Kong, Pokfulam, Hong Kong; 5 USDA ARS Global Change and Photosynthesis Research Unit, Urbana, IL, USA; 6 Department of Natural Resources and Environmental Sciences, University of Illinois at Urbana-Champaign, Champaign, IL, USA; 7 National Center of Supercomputing Applications, University of Illinois at Urbana-Champaign, Champaign, IL, USA; 8 Center for Crop Systems Analysis, Wageningen University, The Netherlands; 9 University of Essex, UK

**Keywords:** Field phenotyping, food security, hyperspectral imaging, photosynthesis, proximal sensing, spectral reflectance

## Abstract

Photosynthesis is currently measured using time-laborious and/or destructive methods which slows research and breeding efforts to identify crop germplasm with higher photosynthetic capacities. We present a plot-level screening tool for quantification of photosynthetic parameters and pigment contents that utilizes hyperspectral reflectance from sunlit leaf pixels collected from a plot (~2 m×2 m) in <1 min. Using field-grown *Nicotiana tabacum* with genetically altered photosynthetic pathways over two growing seasons (2017 and 2018), we built predictive models for eight photosynthetic parameters and pigment traits. Using partial least squares regression (PLSR) analysis of plot-level sunlit vegetative reflectance pixels from a single visible near infra-red (VNIR) (400–900 nm) hyperspectral camera, we predict maximum carboxylation rate of Rubisco (*V*_c,max_, *R*^2^=0.79) maximum electron transport rate in given conditions (*J*_1800_, *R*^2^=0.59), maximal light-saturated photosynthesis (*P*_max_, *R*^2^=0.54), chlorophyll content (*R*^2^=0.87), the Chl *a*/*b* ratio (*R*^2^=0.63), carbon content (*R*^2^=0.47), and nitrogen content (*R*^2^=0.49). Model predictions did not improve when using two cameras spanning 400–1800 nm, suggesting a robust, widely applicable and more ‘cost-effective’ pipeline requiring only a single VNIR camera. The analysis pipeline and methods can be used in any cropping system with modified species-specific PLSR analysis to offer a high-throughput field phenotyping screening for germplasm with improved photosynthetic performance in field trials.

## Teaser

We present a plot-level tool to predict photosynthetic parameters and pigment contents with PLSR analysis of sunlit leaf reflectance pixels to offer high-thoughput field screening for improved photosynthetic performance.

## Introduction

Projected population increase and pressures on land and agricultural resource availability induced by a changing global climate is placing increased demand to secure global food supply in the coming decades (Tilman *et al.*, 2009; Foley *et al.*, 2011). Improving photosynthetic capacity has become a target to enable crop yield increases (Monteith and Moss, 1977; Long *et al.*, 2006; Zhu *et al.*, 2010; Evans, 2013). Inefficiencies in the photosynthetic pathway have inspired research efforts to exploit natural variation in photosynthetic capacity (Lawson *et al.*, 2012), and to improve photosynthetic pathways transgenically (Ort *et al.*, 2015). Thus, crop scientists and breeders face the challenge of characterizing genetic improvements in field trials in a high-throughput manner as a screening tool to identify ‘photosynthetically superior’ germplasm (Furbank and Tester, 2011). While photosynthetic capacity has been successfully estimated from hyperspectral imaging at the ecosystem scale (Serbin *et al.*, 2015), it is often too coarse in spatial resolution to discriminate in mixed germplasm field trails. While hyperspectral analysis has predicted leaf-level photosynthetic capacities and pigment contents (Serbin *et al.*, 2012; Ainsworth *et al.*, 2014; Yendrek *et al.*, 2017; Silva-Perez *et al.*, 2018), it has limitations as leaf clip measurements only pinpoint a few individual leaves in a plot canopy. Currently there are limited tools to screen a whole plot, rather than individual leaves, for photosynthetic performance. Plot-level estimations with proximal sensing platforms are needed to allow rapid capture of reflectance from all sunlit vegetation in the sensor range, eliminating the need to make assumptions about plot performance based on leaf-level samples, and expanding the spatial and temporal capabilities of analysis to capture hundreds of plots in a single day.

The maximum carboxylation rate of Rubisco (*V*_c,max_) and maximum electron transport rate in given conditions (*J*_max_) are widely used as determinants of photosynthetic capacity for the carbon reduction cycle and the electron transport chain, respectively (von Caemmerer and Farquhar, 1981; von Caemmerer, 2000), and are traditionally derived at the leaf level with infra-red gas exchange analysis. The response of leaf-level CO_2_ assimilation to incrementing CO_2_ is measured (Long and Bernacchi, 2003) and analyzed (Sharkey *et al.*, 2007) according to the mechanistic model of photosynthesis (Farquhar *et al.*, 1980). The quantum yield of CO_2_ fixation (ϕCO_2_) and maximum light-saturated photosynthetic rates (*P*_max_) are also used as determinants of photosynthetic operating efficiency, as derived from leaf-level gas exchange measurements of the response of CO_2_ assimilation to incrementing photosynthetically active radiation (PAR) (Ögren and Evans, 1993). Due to the wealth of physiological information provided, leaf-level gas exchange has dominated retrieval of these photosynthetic parameters for decades, but it is limited and time restrictive for the sampling required to measure large crop trials. Additionally, upscaling from leaf gas exchange to determine plot or canopy photosynthetic capacity from gas exchange often requires complex modeling with many assumptions (de Wit, 1965; Evans and Farquhar, 1991; De Pury and Farquhar, 1997; Yin and Struik, 2017; Wu *et al.*, 2019).

Recently, advances have been made in quantifying photosynthesis from spectral analysis at the leaf to ecosystem scales. At the leaf level, with a hand-held spectral leaf gun, photosynthetic capacity (*V*_c,max_ and *J*_max_) and chlorophyll, carbon (C) and nitrogen (N) content have been predicted successfully from hand-held reflectance spectroscopy across the full electromagnetic spectrum (400–2500 nm) for tree species (Serbin *et al.*,, 2012, 2016), productive cropping systems (Ainsworth *et al.*, 2014; Yendrek *et al.*, 2017; Silva-Perez *et al.*, 2018; Ely *et al.*, 2019), and in field trials of *Nicotiana tabacum* with altered photosynthetic pathways (Fu *et al.*, 2019; Meacham-Hensold *et al.*, 2019). Partial least squares regression (PLSR) analysis of reflectance spectra has also been applied to predict photosynthetic capacity with airborne hyperspectral imaging at the agroecosystem canopy scale (Serbin *et al.*, 2015); however, the most advanced satellite hyperspectral systems capture ~1 pixel per 10–30 m (Transon *et al.*, 2018), which is too coarse in spatial resolution to identify genotypic variation within field trials of many small plots. Advanced UAV (unmanned aerial vehicle) systems are able to capture greater spatial resolution (~40 cm per pixel) (Zarco-Tejeda *et al.*, 2013; Ruwaimana *et al.*, 2018), but still fall short of the millimeter resolution required to build models to predict photosynthetic capacities at the scale of individual leaves in small plots. While multispectral cameras are widely available at higher resolution and used to derive plot-level spectral vegetation indices (SVIs) from discreet spectral wavelengths (Curran *et al.*, 1990; Gamon *et al.*, 1992; Thenkabail *et al.*, 2000; Zarco-Tejada *et al.*, 2002; Haboudane *et al.*, 2004), SVIs are not able to determine photosynthetic parameters beyond structural inference on physiological processes from discreet spectral bands. Satellite-mounted multispectral imaging systems have also been widely exploited to derive spectral indices such as the enhanced vegetation index (EVI) and normalized difference vegetation index (NDVI), and, more recently, solar-induced fluorescence (SIF) (Guanter *et al.*, 2014; Porcar-Castell *et al.*, 2014; Guan *et al.*, 2016) and linked to ecosytem gross primary productivity (GPP) (Smith *et al.*, 2002; Wylie *et al.*, 2003; Rahman *et al.*, 2005; Zhang *et al.*, 2014, 2018; Barnes *et al.*, 2017; Shi *et al.*, 2017; He *et al.*, 2019). Multispectral SVI and SIF estimates have been incorporated into terrestrial biosphere models to predict photosynthetic capacities at the ecosystem scale (Demarty *et al.*, 2007; Kattge *et al.*, 2009; Zhang *et al.*, 2014), but have not been used to predict photosynthetic capacity in smaller scale plot trials.

Resolving hyperspectral analysis of photosynthetic parameters at the plot level holds many practical and technical challenges. First, hyperspectral cameras and sensors that capture reflectance at the spatial and spectral resolution required for plot-level analysis are often limiting in terms of availability, affordability, and suitability for field trial scanning. Secondly, field phenotyping proximal sensing platforms (Deery *et al.*, 2014) to house such sensors are not currently commercially available and need to be fabricated for purpose. Thirdly, hyperspectral imaging systems generate memory-intensive three-dimensional data sets with two spatial dimensions (*S*_x_ and *S*_y_) and one spectral (*S*_λ_) dimension, forming ‘hypercubes’ (Bannon, 2009), necessitating advanced data storage systems and custom analysis pipelines. Fourthly, at the plot level, plant geometrical structure, leaf scattering properties, background soil, and dynamic environmental conditions (Verhoef, 1984; Vogelman *et al.*, 1996; Gao *et al.*, 2000; Jay *et al.*, 2016) need to be resolved against leaf-level ‘ground truth’ measurements to accurately infer photosynthetic performance upscaled from leaf to plot level. Finally, ensuring use of this technology answers important physiological questions requires effective interdisciplinary collaboration between engineering, computational, and biological specialists.

In this study, we present a plot-level high-throughput phenotyping platform housing two hyperspectral cameras. One visible near infra-red (VNIR) camera captured reflectance from 400 nm to 900 nm (spectral resolution 2.1 nm) and the second near infra-red (NIR)/shortwave infra-red (SWIR) camera from 900 nm to 1800 nm (spectral resolution 4.9 nm). We created an automated hyperspectral imaging processing pipeline that extracts plot-level sunlit vegetation pixel reflectance spectrum to predict *V*_c,max_, *J*_1800_, chlorophyll content, Chl *a:b*, C content, N content, *P*_max_, and ϕCO_2_. From PLSR analysis of plot-level reflectance spectra from hyperspectral images, we predict these photosynthetic traits in field trials of wild-type and genetically modified lines of *N. tabacum*. We assess the contribution of spectral regions and the applicability of this technique to the field phenotyping community, and offer a tool for high-throughput phenotyping of large-scale crop trials to facilitate screening for increasing crop yields.

## Materials and methods

Data from two growing seasons (2017 and 2018) were used in this study, presented in two performance tests. For performance test 1, three wild-type and seven transgenic *N. tabacum* lines were measured over the 2017 and 2018 growing seasons (Table 1). Measurements in 2017 were taken from 22 June to 1 August and in 2018 on 24 and 25 July. For performance test 2, two wild-type and eight transgenic *N. tabacum* plants were measured in 2018 on 26, 27, and 28 July. In performance test 1, predictive models were built from hyperspectral reflectance (both leaf and plot level) with ground truth data from gas exchange measurement of CO_2_ response curves for *V*_c,max_ and *J*_1800_, and leaf pigment extractions for chlorophyll content, Chl *a:b*, C content, and N content. For performance test 2, predictive models for plot- and leaf-level *P*_max_ and ϕCO_2_ were trained with ground truth data from gas exchange measurement of light response curves.

**Table 1.** *Nicotiana tabacum* genotypes used in this study and description of transgenic modification, with reference to detailed description of transformation

Genotype	Year(s) grown	Transgene	Expected transgene function
**Petite Havana**	2017 and 2018	None (WT)	NA
**Samsun**	2017 and 2018	None (WT)	NA
**Mammoth**	2017	None (WT)	NA
**Single R antisense**	2017	Rubisco small subunit antisense from *Nicotiana benthamiana*. 40% of wild-type Rubisco, background: W38 (Hudson *et al.*, 1992)	Reduced photosynthetic capacity
**Double R antisense**	2017 and 2018	Rubisco small subunit antisense from *Nicotiana benthamiana*. 10% of wild-type Rubisco, background: W38 (Hudson *et al.*, 1992)	Reduced photosynthetic capacity
**Bypass AP3**	2017 and 2018	Two transgenic genes expressing the enzymes glycolate dehydrogenase and malate synthase as an alternative photorespiratory pathway, background: Petite Havana (South *et al.*, 2019)	Increased photosynthetic capacity, by reduction of energy loss associated with photorespiration.
**Bypass AP3/RNAi**	2018	Same as Bypass AP3 but with RNAi to down-regulate native chloroplast glycolate transport, background: Petite Havana (South *et al.*, 2019).	Increased photosynthetic capacity, by reduction of energy loss associated with photorespiration.
**PSBS-43**	2017 and 2018	Increased PsbS mRNA levels from transformation with *Nicotiana benthamiana* Psbs coding sequence and 35S promoter, background: Petite Havana (Głowacka *et al.*, 2016, 2018)	Increased photosynthetic capacity, due to increase in electron transport metabolite pools.
**Psbs-4**	2017 and 2018	Decreased PsbS mRNA levels from transformation with *Nicotiana benthamiana* Psbs coding sequence and 35S promoter, background: Petite Havana (Głowacka *et al.*, 2016, 2018)	Reduced photosynthetic capacity, due to decreased electron transport metabolite pools.
**VPZ-23**	2017 and 2018	Three transgenes from *Arabidopsis thaliana*, expressing violaxanthin de-epoxidase (VDE), zeaxanthin epoxidase (ZEP), and PSII subunit S (psbS), background: Petite Havana (Kromdijk *et al.*, 2016)	Increased photosynthetic capacity, due to overexpressed xanthophyll cycle enzymes.
**LMD**	2018	Transgene from *Arabidopsis thaliana* expressing plastid division protein (FtsZ), background: Petite Havana.	Low mesophyll density: increased chloroplast size and decreased chloroplast number.
**LCD**	2018	Decreased mRNA levels of low cell density (LCD1) homolog of *Nicotiana tabacum* by RNAi, background: Petite Havana.	Low mesophyll cell density and lowered photosynthetic capacity

### Plant material

In 2017, three wild-type *N. tabacum* cultivars and six transgenically modified lines (described in detail in Table 1) were grown at the University of Illinois Energy Farm Facility in Urbana, Illinois (40°03'46.4''N, 88°12'25.4''W, 215 m above sea level). All experiments consisted of four replicated plots of each genotype arranged in a 6×6 grid and spaced 0.38 m apart with 36 plants per plot. Each plot measured ~2×2 m. All transgenic material is expressed in the Petite Havana background, with the exception of the Rubisco antisense lines in the W38 background. Seedlings were germinated in greenhouse conditions in float trays using a coir soil mix (Coco loco) maintained daily at 150 ppm N using a 20–20–20 general-purpose water-soluble fertilizer. Plants were transplanted to the field at the four-leaf stage. High levels of ESN Smart Nitrogen (310 kg ha^–1^, ~150 ppm soil concentration) were applied to the field site 2 weeks prior to transplanting. A broad action herbicide, glyphosate-isopropylammonium (41%) (Killzall; VPG) (15 liters at 70 g l^–1^) was applied once to all plots 2 d prior to transplanting. A biological pesticide *Bacillus thuringiensis* var. *kurstaki* (54%) (DiPel PRO) was applied to the prepared field site 5 d prior to transplant and at biweekly intervals thereafter to control for tobacco pests. Irrigation was provided to all plots as needed to eliminate water limitation throughout growth.

In 2018, two wild-type, five previously grown transgenic lines, and three newly added transgenic lines (described in detail in Table 1) were grown according to the same protocol as in 2017. All transgenic plant material was homozygous, with the exception of the single Rubisco antisense and decreased PsbS line (4-KO). Single Rubisco antisense plants were planted to the field without screening. The 4-KO seedlings were screened 8 d post-emergence with chlorophyll fluorescence imaging to identify and select only plants with the PsbS knockout phenotype for low non-photochemical quenching (NPQ).

### Hyperspectral image collection

**Fig. 1.**
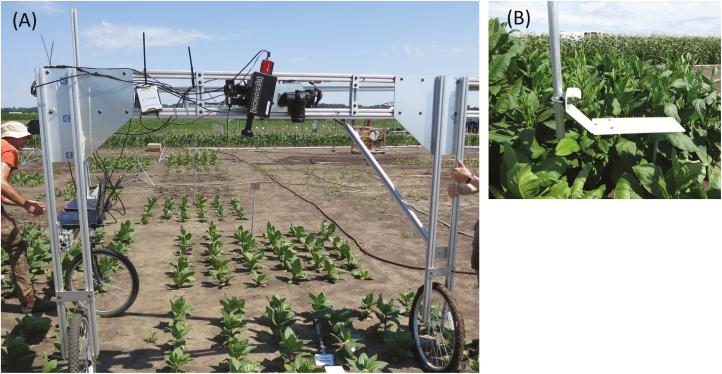
A ground-based phenotyping platform housing two hyperspectral cameras and an RGB camera (A), with a moveable white reflectance panel mounted at the top of the canopy level (B).

A ground-based field phenotyping platform was built to house two hyperspectral push-broom cameras mounted on a horizontal beam (Fig. 1A). The first hyperspectral imaging camera (PIKA II; Resonon, Inc., Bozeman, MT, USA) captured spectral radiation from 400 nm to 900 nm in 2.1 nm contiguous bands (240 spectral bands in total) with 640 spatial channels. The second camera (PIKA NIR: Resonon, Inc.) recorded spectral radiation from 900 nm to 1800 nm in 4.9 nm contiguous bands (164 spectral bands) with 320 spatial channels. Both cameras were mounted at a height of 1.6 m from the soil and were triggered simultaneously above a plot to acquire two images during an ~30 s scan. Images were captured in high-irradiance conditions during a 3 h window around solar noon and stored using SpectrononPro software (Resonon, Inc.). A 99% reflective white Teflon panel was mounted horizontally and level with the top of the plant canopy and captured in the field of view for each image (Fig. 1B). Images were captured and stored in raw data mode. The cameras were calibrated to remove electrical and dark current daily prior to data acquisition. Camera integration time was set at 20% below the saturation point according to the radiance signal from the Teflon panel before each scan to avoid saturation.

### Hyperspectral image analysis pipeline

**Fig. 2.**
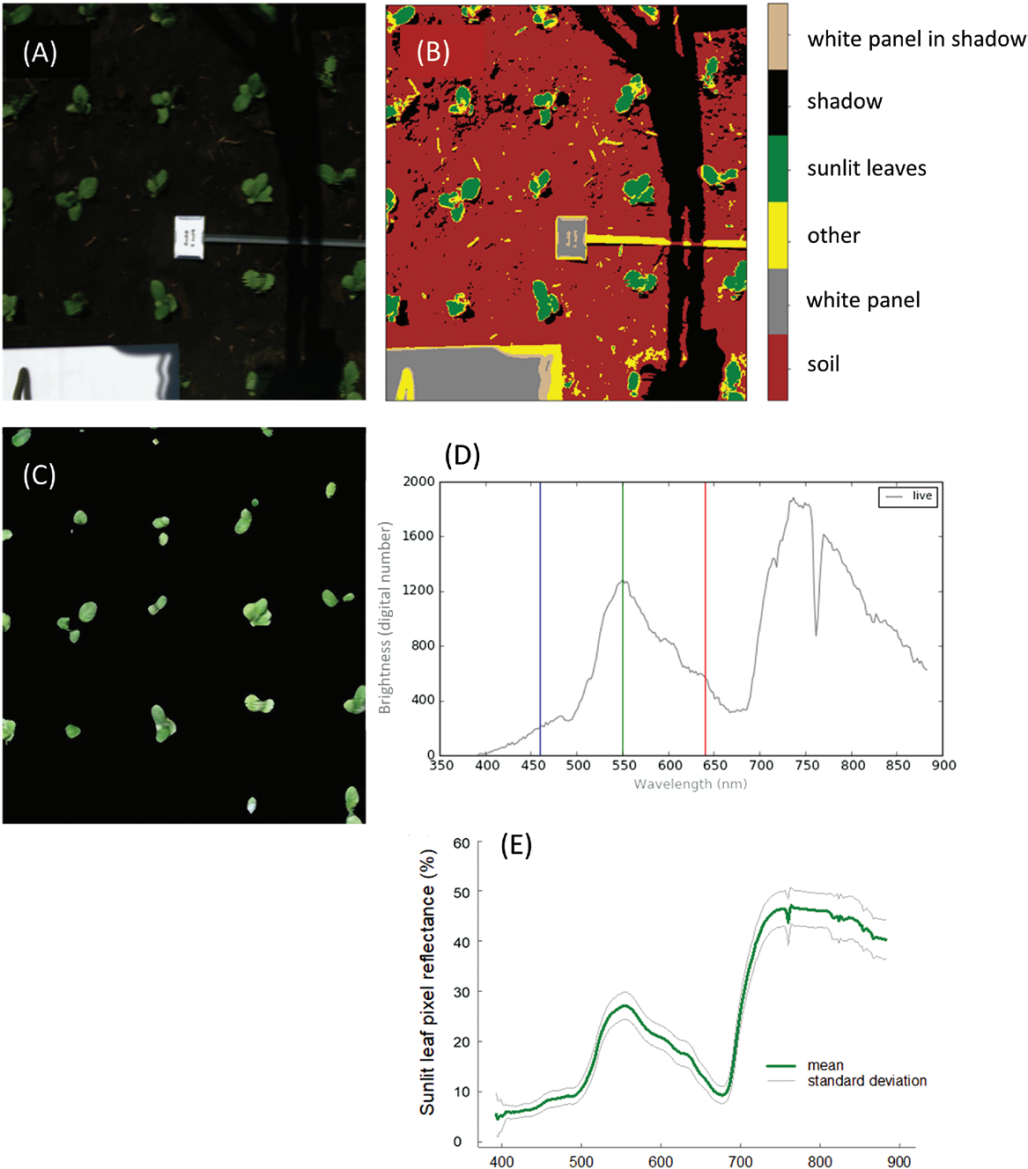
Example of the hyperspectral image analysis processing workflow. Images are captured in raw data mode (digital numbers) and represented as an RBG image (A). Pixels are separated into categories using K-means clustering (B) to extract all sunlit leaf pixels, and converted to reflectance (C) from raw data in digital numbers (D). Mean value and the SD of all sunlit leaf reflectance pixels are computed (D).

An automated image analysis pipeline was created using Python (Python Software Foundation, https://www.python.org/), to extract spectral reflectance from images acquired in raw data mode (Fig. 2A). Data from each camera went through the same three phases of processing: first, conversion of raw data in digital numbers to radiance using radiometric calibration; secondly, the classification of pixels (Fig. 2B); and, thirdly, conversion of radiance pixels to reflectance (Fig. 2C). For the first phase, raw data were converted to absolute radiance using radiometric calibration files from the camera manufacturer. In the second phase, the image was segregated to represent six matter classifications using K-means clustering (Spath, 1985) which separated pixels of interest (sunlit leaves and Teflon) from shaded leaves, soil, platform shadow, and non-biological matter. Thirdly, reflectance (*R*) was calculated using the radiance signature from the Teflon white reference captured in each image against a lab-calibrated Teflon standard using Equation 1:

$$\begin{array}{l} R=\frac{S_{sunlit}}{S_{ref}}*R_{ref} \end{array}$$ (1)

where *S*_sunlit_ is radiance from sunlit leaves, *S*_ref_ is radiance from the Teflon panel, and *R*_ref_ is the percentage reflectance from the lab-calibrated Teflon standard. Brightness in digital numbers was converted to percentage reflectance (Fig. 2D), before reflectance from all sunlit pixels in each image was averaged per plot (Fig. 2E). Spectral reflectance from both cameras in the same plot was joined to give reflectance for sunlit leaves per plot from 400 nm to 1800 nm. Spectra were filtered with a polynomial order of 2 using 11 spectral measurements (nm) as the window length (Savitzky and Golay, 1964). Prior to PLSR analysis, spectral bands below 450 nm and above 1700 nm were removed due to excess noise, and those between 1313 nm and 1440 nm were removed due to atmospheric water absorption (Hill and Jones, 2000; Serbin *et al.*, 2015).

### Leaf spectral measurements

Leaf-level spectral measurements were made using a spectroradiometer (Fieldspec4; Analytical Spectral Devices - ASD, Boulder, CO, USA), with a leaf clip attached to a fiber optic cable. Leaf spectral reflectance was measured *in situ* from 400 nm to 2500 nm with spectral resolution of 3 nm in the visible and NIR spectra (350–1000 nm) and 8 nm in SWIR (1000–2500 nm) spectra. The device houses a radiometrically calibrated light source which was standardized for relative reflectance prior to measurement using a Spectralon (Labsphere Inc., North Dutton, NH, USA) panel for white reference. In 2017, three leaves were sampled per plot and five per plot in 2018. Measurements were made on the last fully expanded leaf, maintaining natural leaf orientation avoiding the leaf midrib and edges. On a single leaf, six reflectance spectra were recorded using the leaf clip attachment in different regions of the same leaf. The six spectra for a single leaf were then averaged to give a mean spectrum per leaf. Each single measurement was the mean of 10 scans at a scan speed of 100 ms. A spectral splice correction was applied to each spectrum to remove heat drift effects that may shift the sensors and align the visible and SWIR sensors to the NIR sensors within the Fieldspec4, using the FieldSpectra package in R according to Serbin *et al.* (2015). For quality control, spectra with abnormally high light levels at 450 nm were excluded from analysis to ensure the leaf clip was properly fastened onto the leaf for each measurement. Spectral samples with a deviation from the mean reflectance >2% were eliminated from analysis along with leaves with fewer than four viable spectra.

Predictive PLSR models for all traits were built at both leaf and canopy levels for comparisons. For plot-level models, the averaged ground truth subsamples (three leaf measurements in 2017 and five leaf measurements in 2018) from each plot were used as input for model building and validation. For leaf-level models, each individual leaf subsample measurement was included as a training data point without averaging per plot.

### PLSR analysis

Predictive models were built for eight traits, following PLS principles (Wold *et al.*, 2001) according to the protocol of Serbin *et al.* (2015), modified for *N. tabacum*. Although in our previous work (Fu *et al.*, 2019), multiple stacked machine learning algorithms showed increased predictability (+5% for *R*^2^) of photosynthetic capacity (*V*_c,max_), we used PLSR only in this work given the ability to derive scaling coefficients across the electromagnetic spectra from this technique, which allow inference of important contributing regions of the spectra for trait prediction. Unlike other predictive algorithms, PLSR coefficient loadings can be calculated to infer the physiological importance of specific spectral bands based on known vegetation spectral properties, and thus can be used to confirm the biological relevance of model builds between different devices and scales.

We used the open-source PLS package (Mevik and Wehrens, 2007) in R (The R Foundation for Statistical Computing, Wien, Austria) to create a linear model of waveband coefficients that account for trait variation in reflectance spectra. The optimal number of components (latent variables: LVs) for each model build was determined from the minimum root mean square error (RMSE) of the predicted residual sum of squares (PRESS) statistic (Esbensen *et al.*, 2002), using a leave-one-out cross-validation (CV) approach that then makes a prediction for the out-of-sample observation (Siegmann and Jarmer, 2015). This prevents overfitting. Models were trained with data pairs of leaf or plot reflectance and a ground truth measurement, and cross-validated with 1000 times random resampling to determine model stability. All model *R*^2^ values presented herein are from this CV. Loading weights indicate known spectral peaks or profiles for each model and are translated to the variable importance in projection (VIP), calculated as the weighted sum of squares of PLS weights (Wold *et al.*, 2001; Farrés *et al.*, 2015).

Temperature corrections were not applied to bring photosynthetic parameters to a standard temperature prior to model fitting; absolute plot temperature was not measured at the time of image capture. As such, all leaf- and plot-level models include temperature variation. No outliers were removed from the predictive models presented.

### Infra-red gas exchange measurements

#### CO_2_ response

Photosynthetic (*A*) versus intercellular CO_2_ (*C*_i_) response curves were collected within 30 min of the leaf spectral measurements on the same last fully expanded leaves to determine *V*_c,max_ and *J*_1800_ for each leaf using a portable leaf gas exchange system with a leaf cuvette (LI-6800; LICOR Biosciences, Lincoln, NE, USA). Four machines were used by four operators to ensure unbiased sampling. Leaf temperature was determined as the mean of three measurements with a hand-held IR gun (FLIR TG54, FLIR^®^ Systems, Inc., Wilsonville, OR, USA). Leaf temperature for gas exchange was set to match this mean leaf temperature prior to each CO_2_ response curve, and relative humidity was set to 65%. PAR was set to 1800 µmol m^−2^ s^−1^, and CO_2_ concentrations were adjusted stepwise over a range of 50–2000 µmol mol^−1^ in set increments as follows: 400, 200, 50, 100, 300, 400, 600, 900, 1200, 1500, 1800, and 2000. Leaves were acclimated to chamber conditions for a minimum of 160 s prior to each *A*/*C*_i_ curve with a minimum and maximum wait time of 160 s and 200 s, respectively, before each individual measurement of a response curve. *V*_c,max_ and *J*_1800_ were determined from these *A*/*C*_i_ curves according to the mechanistic model of photosynthesis (Farquhar *et al.*, 1980) and analyzed using a curve fitting utility developed by Sharkey *et al.* (2007). While light response curves were carried out prior to analysis to determine saturating light intensity as ~1800 µmol m^−2^ s^−1^; we refer to maximum electron transport as *J*_1800_ rather than *J*_max_ to avoid potential false claims of true maximal capacity (Sharkey, 2016). Mesophyll conductance (*g*_m_) was constrained according to values for tobacco at 25 °C reported previously, with temperature dependency incorporated from the linear relationship of *g*_m_ with temperature where *y*= –0.44 + 0.058*x* (Evans and von Caemmerer, 2013).

#### Light response

In performance test 2, to train the *P*_max_ and ϕCO_2_ PLSR models, photosynthetic (*A*) versus irradiance (*Q*) response curves were collected within 30 min of leaf spectral measurements, on the same leaves, with a portable leaf gas exchange system (LI-6800; LICOR Biosciences). All environmental settings matched those for *A*/*C*_i_ response curves (temperature to match ambient, relative humidity 65%), but with CO_2_ set to 400 µmol mol^−1^. Irradiance concentrations were adjusted stepwise over a range of 2000–0 µmol m^−2^ s^−1^ in set increments as follows: 2000, 1800, 1400, 1000, 600, 400, 200, 150, 100, 75, 50, and 0.

Leaf absorption for each genotype was determined using an integrating sphere (LI-1800; LICOR Biosciences) connected to a spectrometer (USB-2000; Ocean Optics Inc., Dunedin, FL, USA) as the mean absorptance of six last fully expanded leaves (Supplementary Table S1 at *JXB* online) measured on the last day of performance test 2 (29 July 2018). *A*/*Q* curves were then corrected for absorbed irradiance (*I*_a_). ϕCO_2_ was calculated as the slope of the relationship between *A* and absorbed irradiance below 150 µmol m^−2^ s^−1^. *P*_max_ was calculated by a non-rectangular curve fit according to Thornley and Johnson (1990) as:

$$\begin{array}{l} P_{max}=\frac{I_{a}+P_{max}-\sqrt{{\left(I_{a}+P_{max}\right)}^{2}-4I_{a\;\;\;}\theta P_{max}}}{2\theta }-\;\;\;R_{d} \end{array}$$

where *P*_max_ is maximum light-saturated photosynthesis, ϕ is quantum yield, *I*_a_ is absorbed irradiance, θ is the curvature factor, and *R*_d_ is the dark respiration rate.

### Chlorophyll, carbon, and nitrogen content

In performance test 1, immediately following each leaf spectral measurement, a 2.01 cm^2^ leaf disc was destructively harvested from each leaf using a cork borer, placed in 2 ml tubes and flash-frozen in liquid nitrogen. To determine leaf chlorophyll (mg m^–1^), one leaf disc from each leaf was incubated in 96% (v/v) ethanol for 24 h at 4 °C. The bleached material and ethanol were mixed (100 µl of solution for each sample) and analyzed with a Synergy 2 photospectrometer (BioTek Instruments, Inc, Winooski, VT, USA) at 470, 649, and 665 nm (Lichtenthaler and Wellburn, 1983). To determine leaf carbon and nitrogen content (%), three more 2.01 cm^2^ leaf discs were destructively harvested, and dried until constant mass, and a subset of ground tissue of known mass (3±0.5 mg) was combusted with oxygen in an elemental analyzer (Costech 4010; Costech Analytical Technologies) and calibrated to %N and %C against an acetanilide standard curve.

## Results

### Physiological and spectral characteristics

**Fig. 3.**
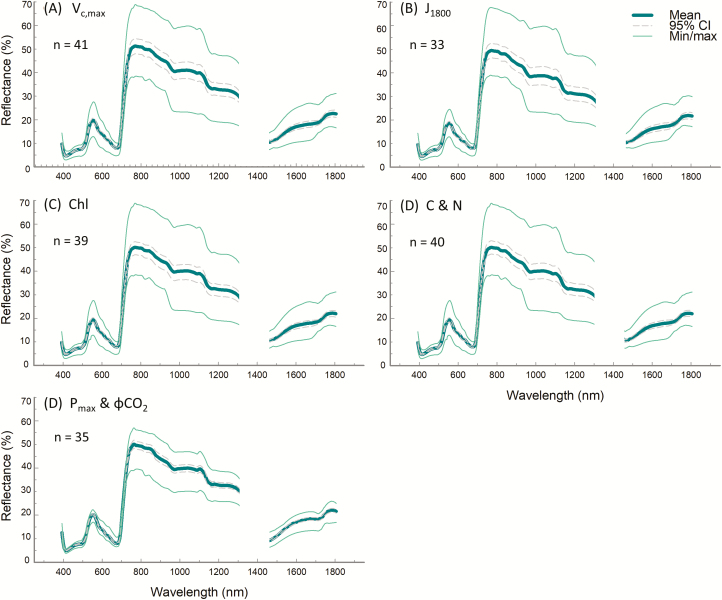
Mean plot-level sunlit leaf reflectance for all spectra included in plot-level PLSR models, from performance test 1, *V*_c,max_ (A), *J*_1800_ (B), chlorophyll content and Chl *a:b* (C), and C and N content (D), and performance test 2, *P*_max_ and ϕCO_2_ (E). Spectra are obtained from our automated image analysis pipeline with the atmospheric water absorption band at 1313–1440 nm removed, and displayed with the minimum and maximum from all data and 95% confidence intervals. *n*=the number of plots the spectra represent. Sample size for each trait varies dependent on the amount of viable ground truth samples taken for each trait.

Our models captured a wide range of natural and genetically altered trait variation over consecutive growing seasons (2017 and 2018). For performance test 1, averaged plot-level measurements of observed *V*_c,max_, *J*_1800_, chlorophyll content, Chl *a:b*, N content, and C content (Supplementary Fig. S1A–F) include variation of environmental and meteorological conditions (between three and five subsamples per plot), with *V*_c,max_ from 13.4 µmol m^−2^ s^−1^ to 359.3 µmol m^−2^ s^−1^ (Supplementary Fig. S1A), *J*_1800_ from 54.9 µmol m^−2^ s^−1^ to 362.1 µmol m^−2^ s^−1^ (Supplementary Fig. S1B), chlorophyll content from 0.1 mg m^–2^ to 0.3 mg m^–2^ (Supplementary Fig. S1C), Chl *a:b* from 1.7 to 3.7 (Fig. S1D), N content from 2.53% to 8.4% (Supplementary Fig. S1E), and C content from 36.2% to 47.4% (Supplementay Fig. S1F). In performance test 2, from light response curves measured between 26 and 29 July in 2018, *P*_max_ ranged between 4.1 µmol m^−2^ s^−1^ and 77.7 µmol m^−2^ s^−1^ (Supplementary Fig. S1G), and ϕCO_2_ ranged between 0.024 µmol m^−2^ s^−1^ and 0.064 µmol m^−2^ s^−1^ (Supplementary Fig. S1H). Hyperspectral reflectance from all sunlit pixels per plot used to build PLSR models for all traits exhibit a peak centering at ~550 nm and high reflectivity in the NIR from 800 nm to 1300 nm, and a smaller peak developing from 1440 nm to 1800 nm, following the expected spectral profile pattern. However reflectance values are slightly lower than expected between 900nm and 1250 nm (Fig. 3).

### Plot-level PLSR predictions

The corresponding reflectance spectrum from all sunlit pixels per plot (Fig. 3) paired with the observed, measured traits (Supplementary Fig. S1) produced robust predictive plot-level models for all traits other than ϕCO_2._ Mean spectra used for each model build in performance test 1 varied slightly, as spectra without a paired ‘ground truth’ sample for each trait were eliminated from model build data sets (Fig. 3). For example, in the SSuD genotype, *J*_1800_ could not be determined from gas exchange as the low Rubisco content meant this genotype was never electron transport limited but instead always Rubisco limited. Given that *J*_1800_ could not be calculated, the *J*_1800_ spectral sample size is reduced compared with the *V*_c,max_ model build (Fig. 3A, B). Similarly, a small number of leaf disc samples for leaf chlorophyll, carbon, and nitrogen content were lost in transportation, storage, or during analysis, creating slight variation in spectral sample used for chlorophyll (Fig. 3C), and C and N (Fig. 3D) model builds.

**Fig. 4.**
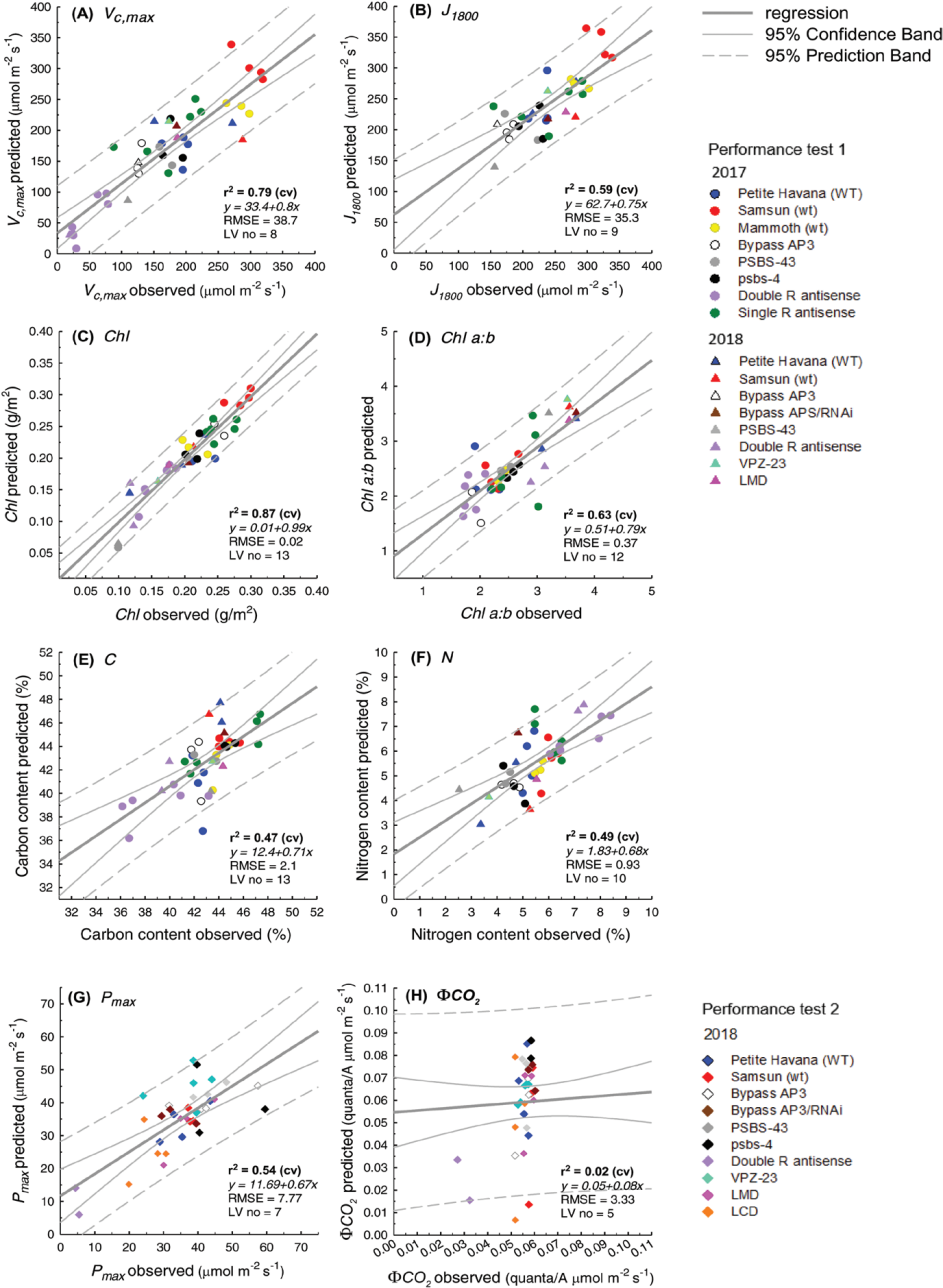
Comparison between observed photosynthetic parameters and those predicted from PLS regression of plot-level sunlit leaf reflectance using a single VNIR hyperspectral camera (450–900 nm) for *V*_c,max_ (A), *J*_1800_ (B), chlorophyll content (C), Chl *a:b* (D), C content (E), and N content (F) in performance test 1, and *P*_max_ (G) and ϕCO_2_ (H) in performance test 2. Observed parameters are the mean of 3–5 leaf-level ground truth measurements, and predictions are the mean of 1000 times cross-validation of the model.

**Fig. 5.**
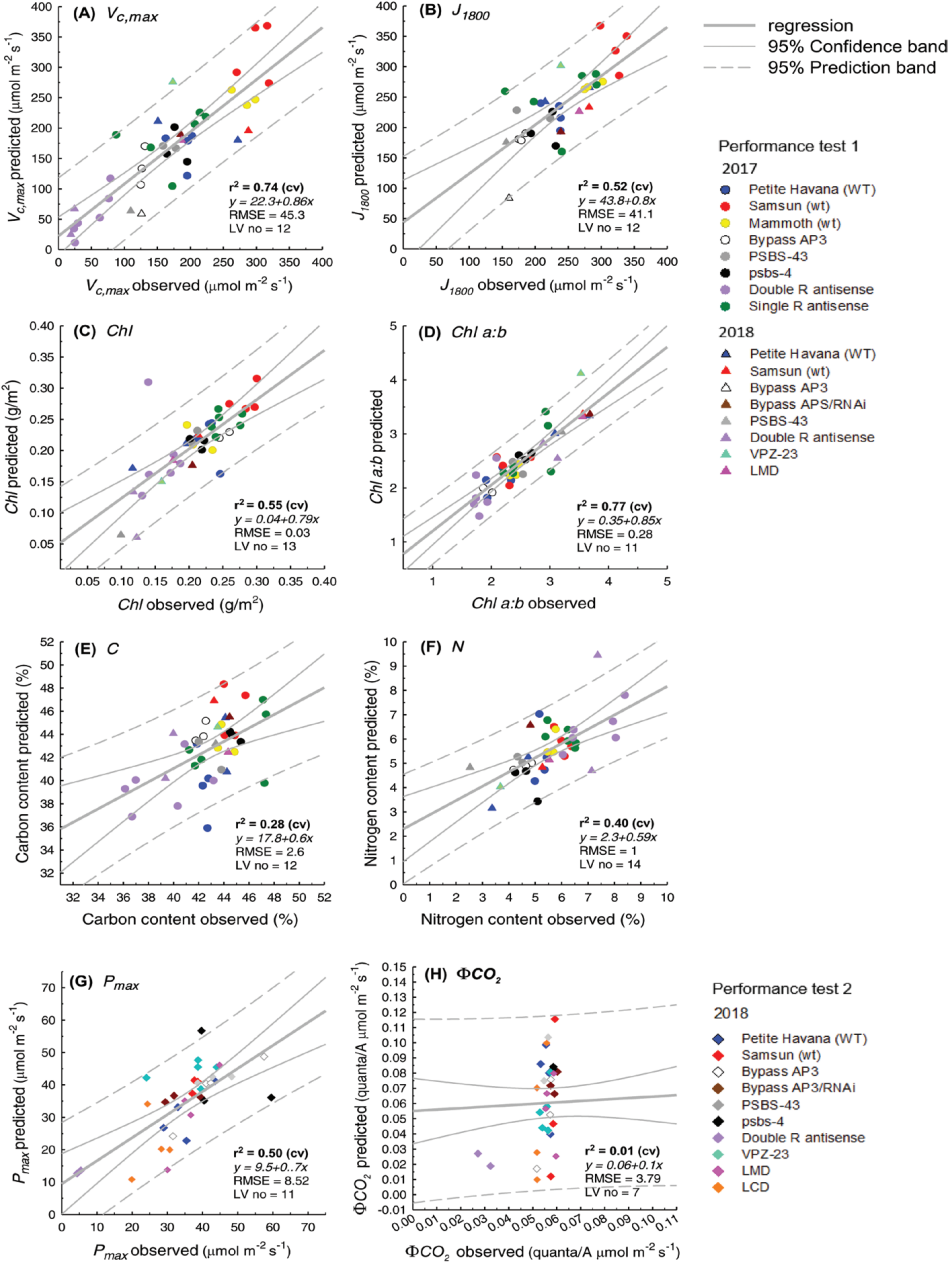
Comparison between observed photosynthetic parameters and those predicted from PLS regression of plot-level sunlit leaf reflectance using both VNIR hyperspectral camera (450–900 nm) and NIR/SWIR (900–1700 nm) cameras for *V*_c,max_ (A), *J*_1800_ (B), chlorophyll content (C), Chl *a:b* (D), C content (E), and N content (F) in performance test 1, and *P*_max_ (G) and ϕCO_2_ (H) in performance test 2. Observed parameters are the mean of 3–5 leaf-level ground truth measurements, and predictions are the mean of 1000 times cross-validation of the model.

Using reflectance spectra from 450–900 nm only, collected with a single VNIR hyperspectral camera, *V*_c,max_ (*R*^2^ 0.79, RMSE% 11.9), *J*_1800_ (*R*^2^ 0.59, RMSE% 11.5), chlorophyll content (*R*^2^ 0.87, RMSE% 10), Chl *a:b* (*R*^2^ 0.63, RMSE% 18.5), and *P*_max_ (*R*^2^ 0.54, RMSE% 10.6) were highly predictable from PLS hyperspectral regression models (Fig. 4; Table 2). PLSR predictions performed moderately well for C content (*R*^2^ 0.47, RMSE% 18.7, Fig. 4E) and N content (*R*^2^ 0.49, RMSE% 15.9%, Fig. 4F), but offered no predictability for ϕCO_2_ (*R*^2^ 0.02, RMSE%, Fig. 4H; Table 2). When compared with the single camera models, PLSR models using both hyperspectral cameras (Fig. 5) had weakened predictive power (lower *R*^2^ and increased RMSE%) for all traits, except Chl *a:b* (Table 2). Using both cameras, *V*_c,max_ (*R*^2^ 0.74, RMSE% 13.1, Fig. 5B), *R*^2^ decreased by 5% and RMSE% increased by 1.9%. However, for Chl *a:b*, predictability increases when both cameras are used (*R*^2^ 0.77, RMSE% 14, Fig. 5D), where *R*^2^ increases by 14%, and RMSE% decreases by 4.5% (Table 2).

**Table 2.** PLSR stability statistics for models built with a single camera (450–900 nm), and for models built with both cameras (450–1700 nm)

Trait	450–900 nm (Pika II)					450–1700 nm (Pika II+Pika NIR)						
	Train *R*^2^	CV *R*^2^	RMSE (trait unit)	RMSE (%)	Bias (trait unit)	Train *R*^2^	CV *R*^2^	RMSE (trait unit)	RMSE (%)	Bias (trait unit)	Change in CV R^2^ (%)	Change in RMSE (%)
*****V*****_**c,max**_ (µmol m^–2^ s^–1^)	0.91	0.79	38.7	11.2	-0.49	0.96	0.74	45.3	13.1	1.64	-5	+1.9
*****J*****_**1800**_ (µmol m^–2^ s^–1^)	0.88	0.59	35.3	11.5	-0.39	0.95	0.52	41.1	13.4	3.42	-7	+1.9
**Chlorophyll** (mg m^–2^)	0.98	0.87	0.02	10	0.002	0.98	0.55	0.03	15	–0.0008	-32	+5
**Chl *a:b***	0.95	0.63	0.37	18.5	0.103	0.97	0.77	0.28	14	0.024	+15	–4.5
**C content** (%)	0.9	0.47	3.1	27.6	0.23	0.91	0.28	2.6	23.1	0.15	-19	+4.4
**N** content (%)	0.85	0.49	0.93	15.5	-0.32	0.95	0.40	1	17	–0.007	-9	+1.2
*****P*****_**max**_ (µmol m^–2^ s^–1^)	0.82	0.54	7.77	1.06	0.12	0.91	0.50	8.52	11.6	0.75	-4	+1
**ϕ** *****CO*****_***2***_ (quanta/A µmol m^–2^ s^–1^)	0.35	0.02	3.33	8325	0.014	0.5	0.01	3.79	9475	-0.099	-0.1	+1150

**Fig. 6.**
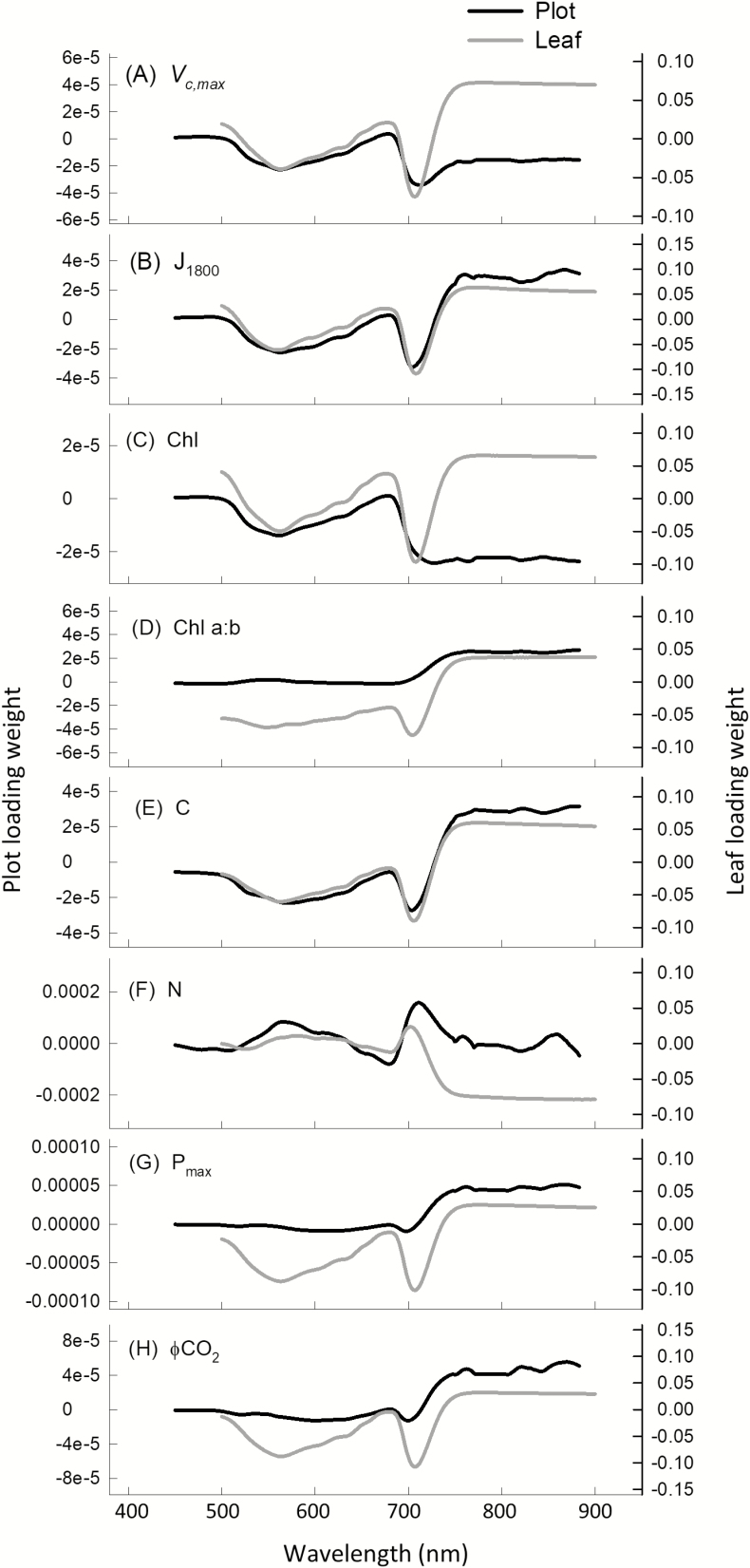
Model loadings from leaf-level and plot-level PLSR models from 450–900 nm for all traits: *V*_c,max_ (A), *J*_1800_ (B), chlorophyll content (C), Chl *a:b* (D), C content (E), and N content (F) in performance test 1, and *P*_max_ (G) and ϕCO_2_ (H) in performance test 2.

**Fig. 7.**
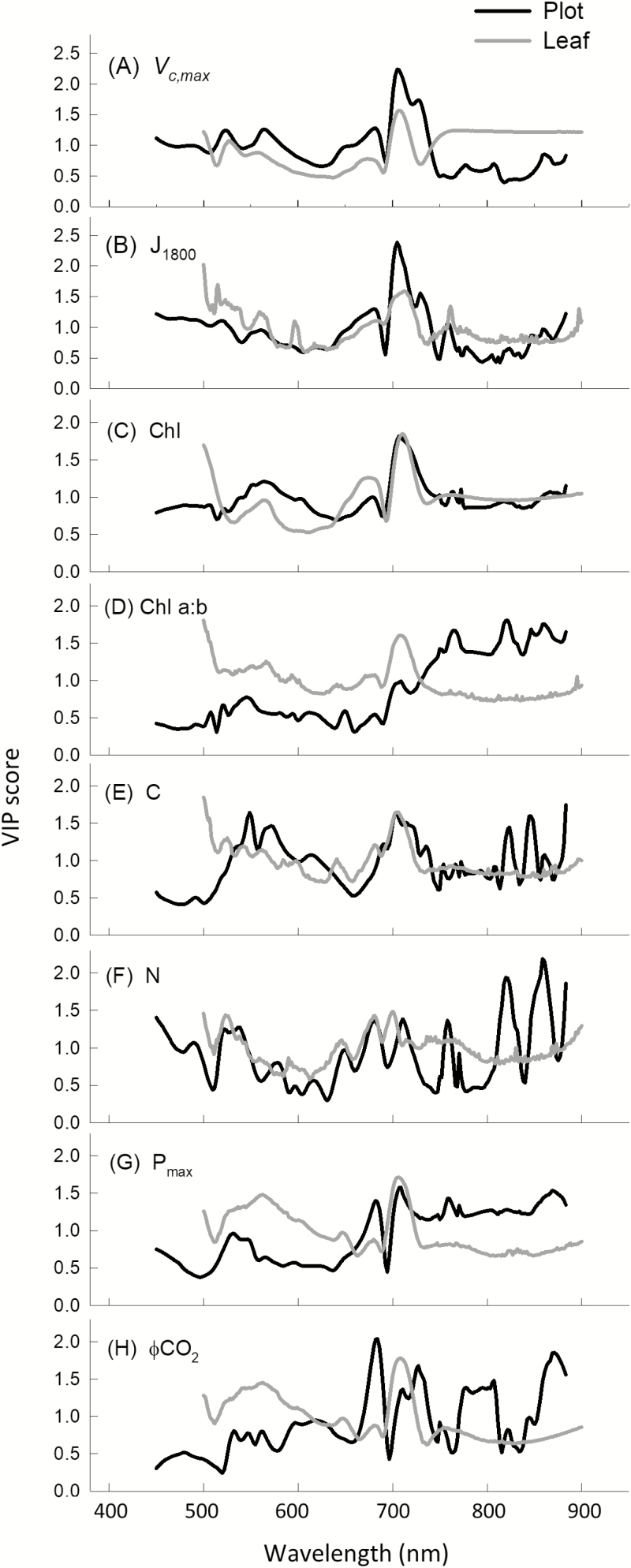
Comparison of variable importance projection (VIP) scores from leaf-level and plot-level PLSR models from 450–900 nm for all traits: *V*_c,max_ (A), *J*_1800_ (B), chlorophyll content (C), Chl *a:b* (D), C content (E), and N (F) in performance test 1, and *P*_max_ (G) and ϕCO_2_ (H) in performance test 2.

Model loading weights indicate the importance of regions of the reflectance spectra for trait variation. For plot-level PLSR predictions with a single VNIR camera (450–900 nm), the region around 700 nm is important for all traits (Fig. 6). When translated to a VIP score for easier interpretation (Fig. 7), 700 nm is shown to be the most important region for *V*_c,max_, *J*_1800_, and chlorophyll content predictions. While ~700 nm is important for all other traits, for C and N content regions from 500 nm to 650 nm and from ~820 nm and ~870 nm in the NIR also hold importance (Fig. 7C, D). For Chl *a:b* and *P*_max_, the entire NIR from 700 nm to 900 nm holds weight. When plot-level model loadings (Fig. 6) and VIP scores (Fig. 7) are compared with those from leaf-level PLSR models built using the same leaves that ground truth the plot-level models, they generally follow the same response pattern for all traits, with the exception of VIP scores for Chl *a:b* (Fig. 7D) and ϕCO_2_ (Fig. 7H).

### Leaf-level PLSR models

When leaf-level PLSR models were built to include different spectral ranges (500–900, 500–1700, and 500–2400 nm), only *V*_c,max_, N content, and *P*_max_ predictability showed minor improvement with greater spectral range (Table 3). The CV *R*^2^ for *V*_c,max_ remained the same when the model used reflectance from 500 nm to 1700 nm, rather than from 500 nm to 900 nm, but there was a 2% increase when the full spectrum was used (500–2400 nm). For *P*_max_ CV, *R*^2^ increased by 7% when the spectral bandwidth matched that of both hyperspectral cameras (500–1700 nm) rather than with the single VNIR only (500–900 nm), but no benefit was seen with the addition of the SWIR (1700–2400 nm). Leaf N content is the only trait for which improved predictability correlated with increased spectral range, with a 3% increase in CV *R*^2^ using 500–1700 nm, and a further 7% increase using 500–2400 nm (Table 2). Unlike the plot-level ϕCO_2_ model, ϕCO_2_ was highly predictable from PLSR analysis of leaf-level spectral reflectance (CV *R*^2^ between 0.61 and 0.63, Table 3).

**Table 3.** PLSR models built at leaf level for all traits using three different spectral ranges (500–900, 500–1700, and 500–2400 nm)

Spectral range (nm)	*R* ^2^ Train	*R* ^2^ CV	RMSECV (trait unit)	RMSE (%)	Model bias (trait unit)	Latent variable (LV) no.
	Vcmax (µmol m–2 s–1)					
**500–900**	0.71	0.67	48.33	13.98	0.066	7
**500–1700**	0.75	0.67	45.21	13.08	0.497	10
**500–2400**	0.79	0.69	41.67	12.06	0.646	11
	***J***_**1800**_ (µmol m^–2^ s^–1^)					
**500–900**	0.59	0.40	38.58	13.38	1.211	11
**500–1700**	0.58	0.39	39.15	13.57	0.454	11
**500–2400**	0.53	0.40	41.38	14.35	0.017	8
	**Chlorophyll content** (mg m^–2^)					
**500–900**	0.82	0.78	0.02	8.82	0.00007	10
**500–1700**	0.78	0.74	0.03	9.76	0.00003	6
**500–2400**	0.80	0.77	0.03	9.32	0.00001	6
	**Chl *a:b***					
**500–900**	0.87	0.78	0.25	8.56	-0.003	14
**500–1700**	0.86	0.79	0.25	8.84	0.0001	15
**500–2400**	0.85	0.76	0.50	7.50	0.005	13
	**C content** (%)					
**500–900**	0.86	0.74	0.96	7.85	-0.011	15
**500–1700**	0.84	0.76	1.01	8.30	0.007	15
**500–2400**	0.86	0.75	0.95	7.84	0.016	15
	**N content** (%)					
**500–900**	0.80	0.66	0.57	8.50	0.011	15
**500–1700**	0.80	0.69	0.58	8.65	0.007	15
**500–2400**	0.85	0.76	0.50	7.50	0.005	15
	***P***_**max**_ (µmol m^–2^ s^–1^)					
**500–900**	0.63	0.50	8.04	10.92	0.12	9
**500–1700**	0.71	0.57	7.15	9.71	-0.04	13
**500–2400**	0.72	0.56	7.04	9.55	0.04	13
	ϕ**CO**_**2**_ (quanta/A µmol m^–2^ s^–1^)					
**500–900**	0.76	0.62	0.004	8.82	0.000004	11
**500–1700**	0.77	0.63	0.003	8.63	-0.000001	12
**500–2400**	0.73	0.61	0.004	9.24	0.000044	9

**Fig. 8.**
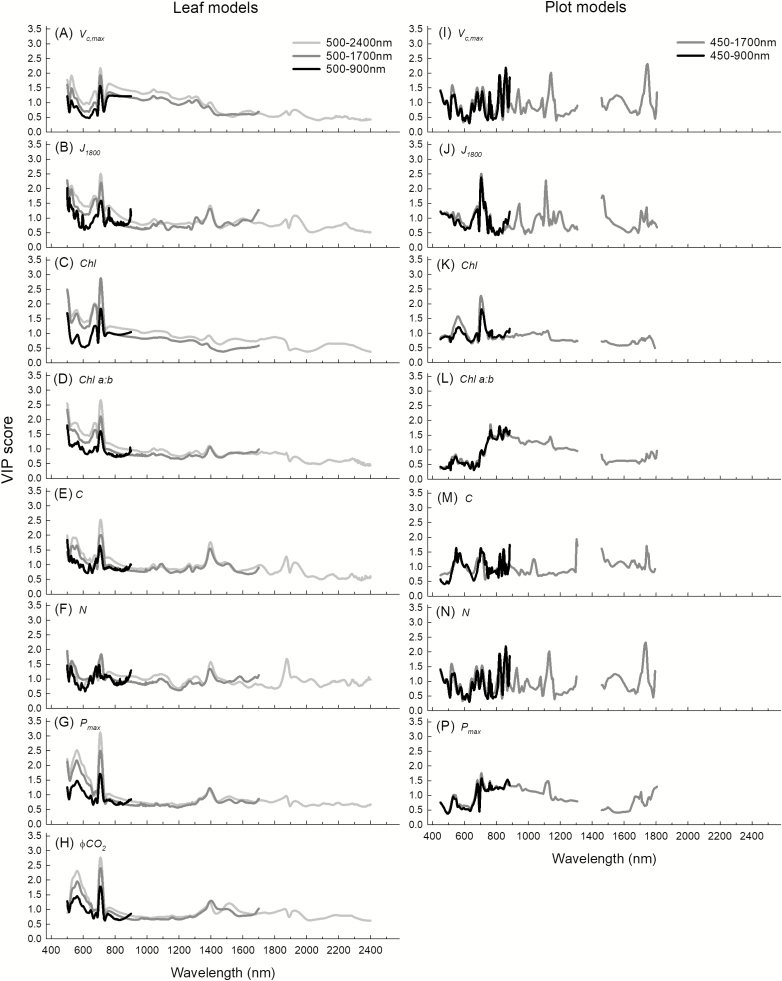
PLSR model variable importance projection (VIP) scores for models built with different spectral ranges for leaf level and for *V*_c,max_ (A), *J*_1800_ (B), chlorophyll content (C), Chl *a:b* (D), C content (E), N content (F), P_max_ (G), and ϕCO_2_ (H), and at the plot level for the same traits, respectively (I–P). VIP scores for plot-level ϕCO_2_ models are not shown due to the lack of predictability of this parameter at the plot level.

For PLSR models built at the leaf level for three different spectral ranges (500–900, 500–1700, and 500–2400 nm, Fig. 8A–H), VIP scores in the VNIR from 400 nm to 800 nm were larger than those in the NIR and SWIR from 800 nm to 2400 nm, for all traits other than leaf C and N contents. For C (Fig. 8E) and N (Fig. 8F) contents, VIP peaks at ~1400 nm and 1900 nm suggest these regions also hold high importance for predictability. Comparing VIP scores for models built with a single VNIR camera (Pika II, 450–900 nm) showed greater variability than those for models built with two cameras (Pika II and Pika NIR, Fig. 8I–P). While all models had high VIP scores between 450 nm and 900 nm, and C and N contents followed a similar pattern seen at the leaf level (Fig. 8M, N), *V*_cmax_ and *J*_1800_ also had VIP peaks at ~1100 nm and 1700 nm (Fig. 8I, J). VIP scores for plot-level ϕCO_2_ models were not shown due to the lack of predictability of this parameter with hyperspectral imaging in this study.

## Discussion

Results show that photosynthetic capacity (*V*_c,max_ and *J*_1800_), maximum light-saturated photosynthesis (*P*_max_), and associated photosynthetic pigment contents (C, N, chlorophyll, and Chl *a:b*) can be predicted using high-throughput proximal plot-level hyperspectral imaging. PLSR analysis of reflectance spectra is now well established as a robust tool for estimating photosynthetic performance at the leaf level (Serbin *et al.*, 2012; Ainsworth *et al.*, 2014; Yendrek *et al.*, 2017; Silva-Perez *et al.*, 2018), and the technique holds integrity when used on plants with altered photosynthetic pathways (Meacham-Hensold *et al.*, 2019). At a broader spatio-temporal scale, data collected with the Airbourne Visible Infrared Imaging spectrometer (AVIRIS) has been used with PLSR analysis of reflectance spectra to successfully predict photosynthetic capacity (*V*_c,max_) at the agroecosystem canopy level, providing lessons for ecosystem and earth system models (Serbin *et al.*, 2015). The results here offer a tool to measure between these contrasting scales to derive photosynthetic capacity as a crop breeding selection tool. The predictive models presented in this study show the utility of hyperspectral imaging as a tool for plot-level phenotyping for superior photosynthetic performance in large-scale field trials, offering potential to screen hundreds of accessions in a single day.

### Spectral compositional features

Electromagnetic energy in the visible range provides the energy for photosynthesis, and absorption in the visible region specifically between 660 nm and 700 nm is of high importance for photosynthetic predictions from reflectance spectra (Serbin *et al.*, 2012; Silva-Perez *et al.*, 2018; Fu *et al.*, 2019; Meacham-Hensold *et al.*, 2019). Similarly, the region of transition from low reflectivity in the visible range to higher reflectivity in the NIR (~750 nm), termed ‘red-edge’, has been utilized to predict *V*_c,max_ (Dillen *et al.*, 2012) and is also heavily weighted in previous PLSR predictive model loadings (Yendrek *et al.*, 2017; Silva-Perez *et al.*, 2018; Meacham-Hensold *et al.*, 2019). These relationships are underpinned by the importance of chlorophyll, nitrogen, and Rubisco in photosynthetic processes (Evans, 1989) and the dominating influence of these pigments on reflectance spectra from 500 nm to 800 nm (Curran, 1989; Elvidge, 1990; Ustin *et al.*, 2009). VIP scores quantify the contribution of each variable (spectral bands) to overall variance and, in this study, when models were built using data from a single VNIR camera (450–900 nm), the greatest peaks in VIP scores are also in the chlorophyll absorption bands and the red-edge regions for *V*_c,max_ and *J*_1800_, *P*_max_, chlorophyll content, and N content (Fig. 7), fitting with previous spectral reflectance compositional observations (Farrés *et al.*, 2015).

Previous leaf-level studies show that some regions of the lower energy NIR, particularly ~1400 nm, are also important for photosynthetic PLSR predictions (Yendrek *et al.*, 2017). However, in this study, plot-level models built using reflectance in the VNIR range only (450–900 nm) give greater predictability than those using reflectance from a greater spectral range (450–1700 nm) (Figs 4, 5; Table 3). This was unexpected and may be the result of compounding factors. In our plot-level analysis using both cameras (450–1700 nm), we removed reflectance between 1313 nm and 1440 nm given convolution of reflectance spectra in that region from atmospheric water absorption properties (Hill and Jones, 2000; Serbin *et al.*, 2015), where removal of these bands is unnecessary when using a leaf clip with an artificial light source. Thus, it follows, with the absence of reflectance at ~1400 nm, that the spectral region detected by the single VNIR camera (400–900 nm) captures the most important regions for photosynthetic predictions. This offers one possible explanation for the strength of PLSR predictions for all parameters in this study from the single VNIR camera (Fig. 4). In addition, when reflectance spectra from both cameras (450–1700 nm) were used to build predictive models, VIP scores for chlorophyll content (Fig. 8K), Chl *a:b* (Fig. 8L), and *P*_max_ (Fig. 8P) show that reflectance from the NIR above 900 nm holds little or no importance (Fig. 8). This is not surprising given that the absorption of chlorophyll occurs in the visible range (Ustin *et al.*, 2009) and that *P*_max_ should be highly related to pigment and pigment pool distributions. However, for *V*_c,max_ (Fig. 8I), *J*_1800_ (Fig. 8J), C content (Fig. 8M), and N content (Fig. 8N), while VIP peaks between 400 nm and 900 nm dominate, peaks at ~1150 nm and 1750 nm are present, suggesting secondary importance of these regions. Despite the known spectral properties for N and C contents in these regions (Curran, 1989; Asner and Martin, 2008), and similarly high VIP scores around ~1100 nm in predictions of *V*_c,max_ from airborne spectroscopy (Serbin *et al.*, 2015), models for these three parameters built with reflectance from both cameras (450–1700 nm) rather than just the VNIR (450–900 nm) are weaker (Figs 4, 5; Table 2).

Chl *a:b* is the only trait for which predictions improve when two cameras (450–1700 nm) are used for the model build rather than the single VNIR (450–900 nm) camera (Figs 4D, 5D). With known chlorophyll absorption dominant only in the visible range, supported by the low loading values for the leaf level Chl *a:b* models above 900 nm (Fig. 8D), this raises questions as to the cause of improved predictability when adding reflectance spectra above 900 nm. This is probably due to the dilution effect for spectral regions of physiological importance when a ratio of two physiological traits is presented. While the Chl *a:b* model is unlikely to be overfit given the reliance on the PRESS statistic in latent variable number selection, physiological importance is reduced, allowing ‘statistical’ number training rather than physiologically based ‘trait’ training. Thus care should be taken to eliminate spectral regions shown to hold little weight for the original trait pair when using this PLSR technique to predict ratio values.

### Leaf-level comparisons

In attempts to understand the relationship between spectral range and predictability power of PLSR models, we built leaf-level models for all of the plot-level ground truth material measured in this study at three different spectral ranges (Table 3). For each trait, we built models first using reflectance spectra measured with the Fieldspec4 from 500 nm to 900 nm, secondly from 500 nm to 1700 nm, and thirdly from 500 nm to 2400 nm. At the leaf level, with a single device measuring from 400 nm to 2500 nm and an artificial light source, the only trait prediction that improved with greater spectral range inclusion was leaf N (500–900 nm CV *R*^2^=0.66, 500–1700 nm CV *R*^2^=0.69, 500–2400 nm CV *R*^2^=0.76, Table 3). The predictability of all other parameters was not increased with increased spectral range. This may be due to the almost equal importance of VIP peaks around 1400 nm and 1900 nm when compared with the chlorophyll and red-edge regions from 500 nm to 800 nm for N content (Fig. 8F). In contrast, at the leaf level for all other predicted traits in this study, the highest VIP scores occur between 500 nm and 800 nm, with only small peaks in the NIR and SWIR (Fig. 8A–H), which may explain the lack of correlation between PLSR prediction power and spectral range included in the leaf-level model builds for *V*_c,max_, *J*_1800_, chlorophyll content, Chl *a:*b, C content, *P*_max_, and ϕCO_2_. While ϕCO_2_ is not predictable with PLSR analysis at the plot level (Figs 4H, 5H), it is highly predictable at the leaf level (Table 3; Supplemenetary Fig. S2), highlighting the need for high variation in observed trait values, to cover greater ‘trait space’ (Ely *et al.*, 2019) for building robust models (Meacham-Hensold *et al.*, 2019). Where observed leaf traits are averaged (between three and five subsamples) at the plot level for ϕCO_2_, observed measurement repetitions are thus reduced, shrinking the trait space and consequently the model prediction strength.

Vegetative structural reflective properties and the comparative loading and VIP scores for leaf and plot-level models from 450 nm to 900 nm (Figs 6, 7) support the strength of plot-level models built with a single VNIR camera (Fig. 4). Loadings and VIP scores may support a lack of improved predictability when models for the same traits are built with reflectance from two cameras that span a greater spectral range (400–1700 nm), but they do not explain the apparent reduction in predictive power (Table 3). N and C content predictions, in particular, should perhaps be improved when lower energy regions of the NIR are included in analysis with both cameras, due to the known absorption features properties of C and N in the NIR (Curran, 1989), and the strong VIP peaks at ~1100 nm and 1700 nm (Fig. 8M, N). This unexpected reduced model strength with increased spectral range is likely to be due to instrumentation limitations. Hyperspectral imaging equipment for phenotyping in field trials is limited. We used two hyperspectral cameras, with different spectral resolution (Pika II, 2.1 nm; Pika NIR, 4.9 nm), different spatial resolution (Pika II, 7.4 µm pixel size; Pika NIR, 30 µm pixel size), and different signal to noise ratios (Pika II,198; Pika NIR, 1885), given the lack of affordability and availability of a single sensor to cover the full electromagnetic spectra. The NIR camera has greater intrinsic error.

### Improving plot-level hyperspectral predictions

The quality of the signal from the Pika NIR (900–1800 nm) camera presents a key challenge throughout this work. Model predictions using two cameras are probably weakened due to technical limitations rather than lack of importance of particular NIR spectral regions for physiological trait prediction. The reflectance profile from the Pika NIR imaging system, ~900–1250 nm, is lower than expected when compared with reflectance measured with a leaf clip. Working with spectral reflectance measured by imaging systems using sunlight rather than a leaf clip with an artificial light source presents challenges, with light having been influenced by the atmosphere before reaching the leaf and again after reflection before detection by a sensor. This results in a more complex signal compared with reflectance from integrated full-spectrum leaf-level devices. For example, quantification of leaf angles, removal of background noise from scattered reflectance at lower canopy levels, removal of background noise from soil (Verhoef, 1984; Gao *et al.*, 2000), and correction for plot temperature at the time of image capture (Serbin *et al.*, 2015) could all improve plot-level model strength. Our plot-level reflectance spectra are also lower between 900 nm and 1250 nm than those from aircraft and other proximal hyperspectral imagers. Proximal hyperspectral imagery usually presents data captured from nadir sensors rather than push-broom scanning platforms. At the time of our data collection, for mounting ~1 m above the target vegetation on a proximal sensing push-cart, push-broom line sensors offered the greatest spatial resolution and affordability. However, the camera angle rotation increases directional anistropy and, coupled with light scattering from background vegetation, increases our signal to noise ratio. While our automated analysis pipeline (Fig. 3) very accurately accounts for radiance at the time of image capture using a Teflon reference panel for accurate conversion to reflectance (Fig. 2B), the signal could probably be improved with an updated nadir scanner and future incorporation of more complex radiative transfer modelling to account for background scattering. Leaf-level VIP scores show less variation than plot-level scores (Fig. 8), particularly in the NIR. While VIP scores are higher at the plot level, peaks do follow the same trends, thus the variation is likely to be indicative of scattering detected by the NIR hyperspectral camera and sensor noise rather than a need to question the true importance of these regions for prediction of a given trait.

The variation in plot-level ground truthing also presents a known challenge as plot-level estimations are trained with leaf-level measurements. While currently this is the only realistic ground truth method for canopy photosynthetic measurements, it is not ideal given the known limitations of applying leaf-level measurements to canopy estimations (Amthor, 1994; Baldocchi and Harley, 1995; De Pury and Farquhar, 1997; Wu *et al.*, 2016) and the known variation in photosynthetic rates and capacities within crop canopies of the same germplasm and even within plant crowns at the highest levels of a canopy due to variation in light environment (Niinemets, 2007). More robust plot-level models could be trained with a greater number of ground truth samples, but the time taken to obtain gas exchange measurements of photosynthetic capacity is a limitation. These challenges persist for the high-throughput phenotyping and the remote-sensing community and, as equipment develops and sensor integration capabilities advance, predictive models of the nature presented in this study will probably improve. Despite these challenges, this study proposes robust plot-level predictions of key photosynthetic parameters and structural traits that are the focus of current research efforts to increase crop yields for global food security (Evans, 2013; Ort *et al.*, 2015).

The challenges facing agricultural production in the face of resource limitation and changing climate necessitates methods for rapid screening of large field trials for productivity and performance. The results from the automated hyperspectral image analysis pipeline we present synthesize high-resolution plot-level information to a single sunlit plot leaf reflectance spectrum for use in a variety of applications. Photosynthetic predictions from PLSR analysis of this output offers a tool for rapid field phenotyping for photosynthetic performance. Such synthesis of large spatial and temporal data sets with user-friendly analysis pipelines that derive biologically relevant outcomes will be increasingly important in the fight for increased global food production. The success of predictive models with a single VNIR hyperspectral camera widens the relevance and potential application of this technique for greater utility, as reduced spectral bandwidth equates to reduced cost of acquisition and operation of hyperspectral imaging systems.

## Supplementary data

Supplementary data are available at *JXB* online.

Table S1. Leaf absorption values used to correct *P*_max_ and ϕCO_2_ for genotypes in performance test 2.

Fig. S2. ‘Ground truth’ *V*_c,max_, *J*_1800_, chlorophyll content, Chl *a:b,* N content, C content, *P*_max_ and ϕCO_2_ values used to train predictive models.

Fig. S3. Comparison between observed photosynthetic parameters and those predicted from PLS regression of leaf-level reflectance using ASD Fieldspec4 with leaf clip attachment for ϕCO_2_ from reflectance from 500 nm to 900 nm and from 500 nm to 1700 nm.

Dataset 1. Spectrum collected with hyperspectral imaging cameras used for model builds for each trait as presented in Fig. 3.

Dataset 2. PLSR model predictions from a single VNIR hyperspectral camera (450–900 nm), as shown in Fig. 4.

Dataset 3. PLSR model predictions from two hyperspectral cameras (450–1800 nm), as shown in Fig. 5.

eraa068_suppl_Supplementary_Dataset_S1

eraa068_suppl_Supplementary_Dataset_S2

eraa068_suppl_Supplementary_Dataset_S3

eraa068_suppl_Supplementary_Table_S1_Figures_S1-S2

## Abbreviations

ϕCO_2_: quantum yield of carbon fixation
*J*_1800_: maximum electron transport rate at 1800 µmol m^−^^2^ s^−^^1^ PAR
*J*_max_: maximum electron transport rate
NIR: near infra-red
PLSR: partial least squares regression
*P*_max_: maximum light-saturated photosynthetic rate
SIF: solar-induced fluorescence
SVI: spectral vegetation index
SWIR: shortwave infra-red
*V*_c,max_: maximum carboxylation rate of Rubisco
VIP: variable importance projection
VNIR: visible near infra-red
