# Supplementary material for: Plot-level rapid screening for photosynthetic parameters using proximal hyperspectral imaging
Source: J Exp Bot. 2020 Feb 24;71(7):2312–28. doi: 10.1093/jxb/eraa068 (PMC7134947; doi:10.1093/jxb/eraa068)
Supplement: eraa068_suppl_Supplementary_Table_S1_Figures_S1-S2 [file eraa068_suppl_supplementary_table_s1_figures_s1-s2.pdf]

Table S1. Leaf absorption values used to correct P_{max} and ϕCO_2 for genotypes in performance test 2.

Genotype	Mean leaf absorption
Petite Havana	0.891 ±0.0058
Samsun	0.895 ±0.004
Double R antisense	0.823 ±0.0046
Bypass AP3	0.892 ±0.011
Bypass AP3/RNAi	0.882 ±0.025
PSBS 43	0.854 ±0.029
psbs 4	0.876 ±0.006
VPZ-23	0.884 ±0.0006
LMD	0.875 ±0.0054
LCD	0.869 ±0.0058

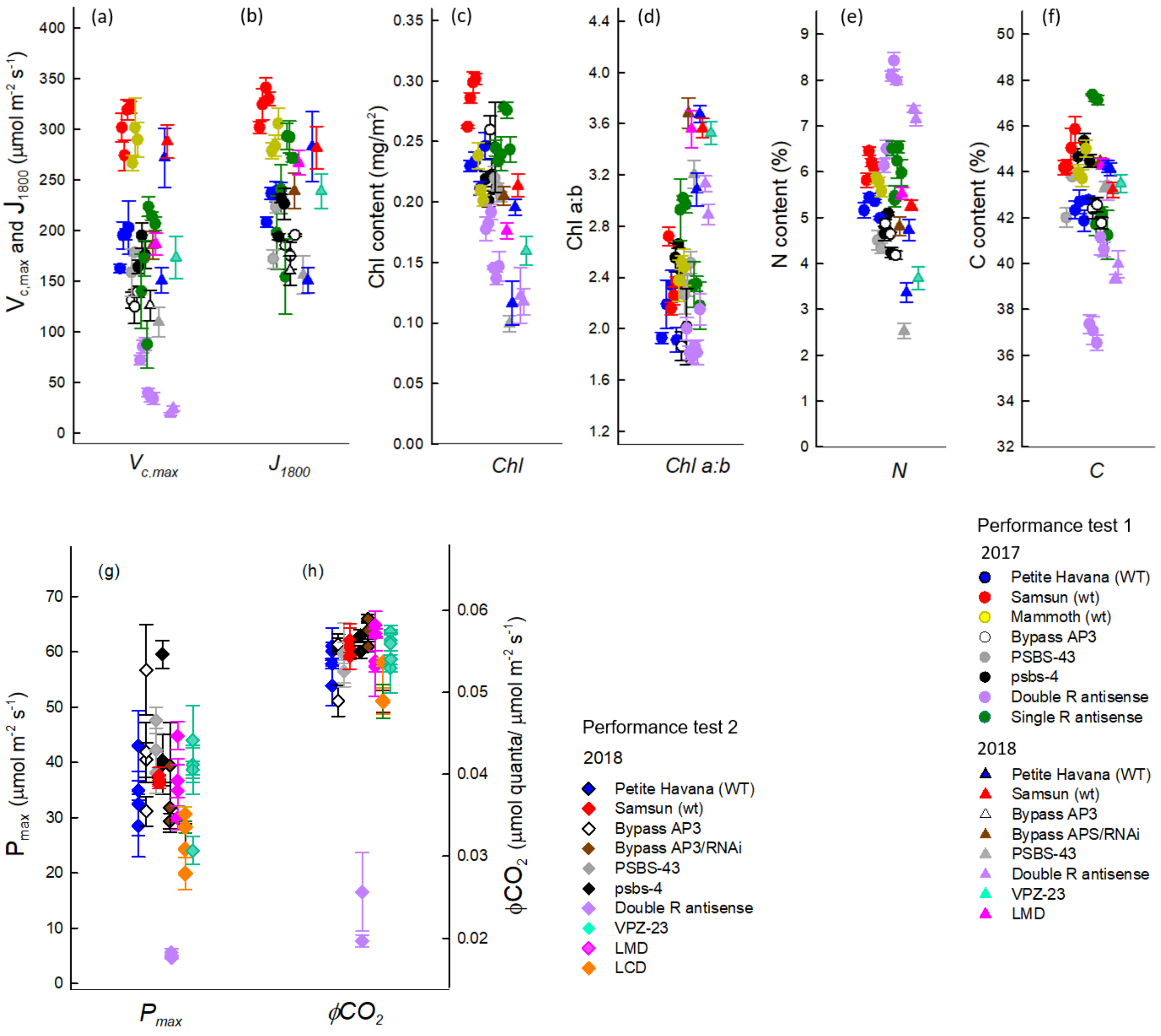

Figure S1

'Ground truth' values used to train predictive models. Each data point represents mean values for each plot included in predictive model build training. On the x-axis, each data point represents mean values for each plot included in predictive model build training. For preformance test 1, $V_{c,max}$ (a) and J_{1800} (b) were calculated as mean value from between 3-5 leaf-level gas exchange measurements from last fully expanded leaves in each plot. *Chl* (c) and *Chl a:b* (d), *N* (e), *C* (f), were calculated from destructive sampling of leaf material for each parameter from between 4-5 last fully expanded leaves in each plot. For performance test 2, P_{max} and ϕCO_2 were calculated from light response curves. Error bars display standard deviation of the subsamples in each plot.

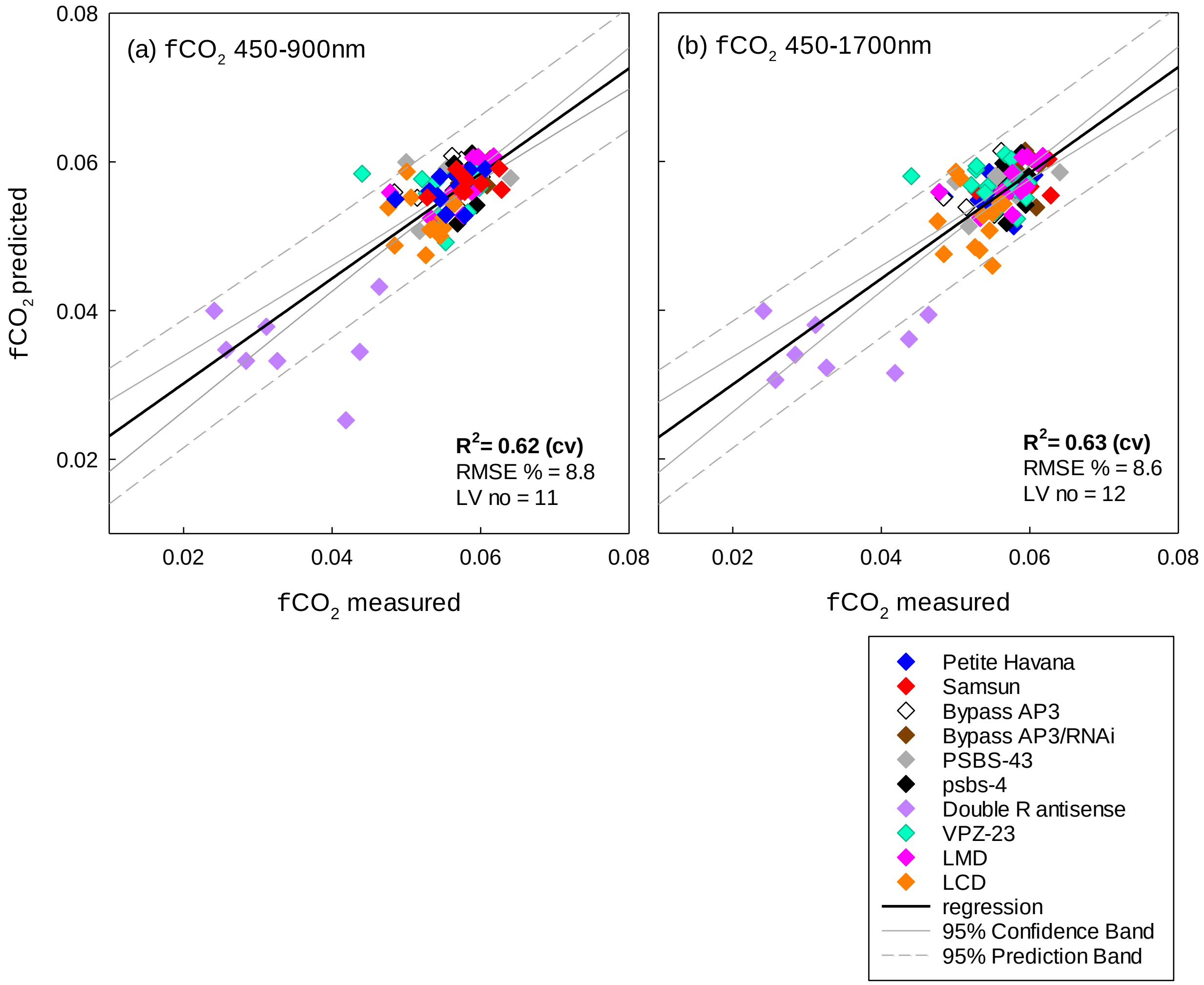

Figure S2

Comparison between observed photosynthetic parameters and those predicted from PLS regression of leaf level reflectance using ASD Fieldspec4 with leaf clip attachment for ϕCO_2 from reflectance from 500-900nm (a) and 500-1700nm (b).
